# Patient and Veteran Engagement in Health Research: the Emergence of a Field of Study

**DOI:** 10.1007/s11606-022-07393-9

**Published:** 2022-03-28

**Authors:** Susan L. Zickmund, Dominick L. Frosch, Kristin L. Carman

**Affiliations:** 1 IDEAS Center of Innovation, VA Salt Lake City Health Care System; Department of Internal Medicine, University of Utah, Salt Lake City, UT USA; 2 Health Science Diligence Advisors, LLC, Redwood City, CA USA; 3 Patient-Centered Outcomes Research Institute, Washington, DC USA

There is increased academic interest in patient engagement in the health research process. A quick search using PubMed indicates that patient engagement literature citations grew over 900% from 2000 to 2020. This growth stems in part from the 2010 establishment of the Patient-Centered Outcomes Research Institute (PCORI), its reauthorization in 2019, and the inclusion of engagement as fundamental to all PCORI’s work, including research awards.^1^ In line with these efforts, the Veteran Administration (VA’s) Health Services Research and Development (HSR&D) in 2015 established a national workgroup to outline an approach to local Veteran engagement initiatives and to develop goals for promoting Veteran research engagement nationally. This *JGIM* supplement on patient and veteran engagement in health research reflects the shared goals of PCORI and VA HSR&D to assess what we have learned about engagement in research and inform a larger strategy to continue developing the science of engagement.

The work of PCORI and VA HSR&D has built on many fields, including community-based participatory research (CBPR), that inform how to structure and support engagement of partners in research. After a decade of supporting patient-centered outcomes research, hundreds of research teams have implemented patient and stakeholder engagement as part of the requirements for PCORI and VA HSR&D funding.

PCORI has generally been nonprescriptive about how engagement occurs in its funded studies, only that it must occur, and provides guidance to support engagement.^2^ This reflected the lack of a robust evidence base to guide the conduct and understand the outcomes of engagement on research, individuals, and more distal outcomes like uptake and use of interventions. Similarly, the efforts by VA HSR&D led to a substantial growth in Veteran engagement activities, with many of the perspectives and original articles in this supplement resulting from this investment.^3,4^ The shared emphasis on engagement has also helped fuel the growth of engagement in other funding agencies, including the Department of Defense (DOD) and the National Institutes of Health (NIH).

The scale of the work by PCORI, the VA, and other funders has helped to stimulate the burgeoning field of the *science* of engagement—that is, the systematic study of methods for and outcomes of engagement to inform high-quality, patient-centered health research. During the early years of PCORI’s funding, a modest number of methods program awards were funded to advance methods of engaging patients and other stakeholders in research. While there have been modest investments in research, much of this burgeoning knowledge about the benefits, frameworks, and models for how to engage has proliferated based on more applied and experiential descriptions of engagement rather than systematic comparisons of varying approaches.

## WHAT WE DISCOVERED FROM THIS SUPPLEMENT

Most evident to these guest editors was the outpouring of interest in our engagement supplement. As noted, over 80 papers were submitted in response to our call for papers. Also striking is the wide range of studies and submitting institutions, all with different designs and goals. Two key funders, PCORI and VA, funded the vast majority of projects described in the submissions we received, demonstrating the impact of systematic institutional emphasis on engagement. The articles submitted and the articles included demonstrate the substantial commitments both PCORI and the VA have made in catalyzing engagement in research.

The perspectives and original research articles in this supplement share a focus on the lived experience of the participants of engagement, as well as exploring barriers and facilitators that can affect group cohesion.^5–15^ Many of the studies measured satisfaction with the engagement process as a key outcome. One strong commonality amongst the perspectives and original research articles is the use of descriptive approaches, often outlining the temporal flow of the engagement process for a project and frequently accompanied by qualitative and/or mixed methods.

A deep dive into the pieces in this supplement shows the diversity of literature cited, rather than the same seminal articles. What is highlighted in these perspectives and original articles is that many study teams across different topics and institutions are trying to understand how best to capture the engagement process and participants’ experiences and improve engagement. The innovative ways the teams found to capture and promote engagement activities can serve as a repository for creative practices that are occurring in the field.

From this supplement, we also conclude that engagement is a field coming into its own and trying in real-time to discover central tenants, principles, models, and approaches. This supplement provides a snapshot in time of a field of study emerging into existence. This journal issue amplifies that engagement of patients and stakeholders is occurring across health care research and that it is dynamic and multifaceted. Through this research, we are learning more about how engagement can affect the study’s conduct by shaping the user’s orientation, acceptability, feasibility, quality, and the relevance of research, as well as the challenges to engagement. Within the pages of this supplement are myriad examples of documenting and measuring the engagement process. Multiple tools, surveys, and other materials are collected in this supplement that can aid engagement efforts of other investigative teams and, therefore, help move the field forward.

## TO EFFECTIVELY GROW THE FIELD: LESSONS LEARNED FROM THE SUPPLEMENT

Lessons learned from this supplement include two key points: the need to strengthen the engagement community as well as the need to diversify and strengthen the science of engagement itself. The sheer number of approaches and frameworks included in this supplement highlights the immense creativity and the diversity of activities used. Multiple teams faced the daunting task of creating an environment ripe for engagement and assessing its impact. This supplement also reveals the progress that still needs to occur to forge a more unified intellectual community.

We are struck by the parallels between the current state of engagement and the development of the field of implementation science. Around the turn of the millennium, both NIH and HSR&D confronted the long pipeline needed to advance innovations in health sciences into clinical practice. Both NIH and the HSR&D via the Quality Enhancing Research Imitative (QUERI) used workshops, trainings, and targeted funding initiatives to train the generations of implementation scientists. The emergence of the journal *Implementation Science* helped foster a more unified community of implementation scientists through a shared publication that has the goal of advancing how best to foster the uptake of proven health innovations.

In a similar way, we can see parallels to early implementation science based on the perspectives and original articles in this engagement supplement. These articles represent the experiences of multiple teams applying a diversity of approaches to the common cause of promoting and measuring successful engagement. A similar push for nationwide trainings, workshops, and targeted funding mechanisms can further unite the field and help create widely accepted practice patterns and gold standards. Such systematic approaches can help answer the essential questions: what does engagement accomplish, why is it important, and how can it best be supported and accelerated? From such an investment, the next generation of scholars in the engagement community will emerge, furthering the construction of an engagement community. To realize the above efforts will require agencies to be willing to fund engagement studies to test out effective engagement designs, measurements, and outcomes, including more proximal impacts on the individuals participating, conduct of the studies, and the more distal aims of improving health care and health.

Despite our growing body of evidence, we do not yet have answers to critical questions. Substantial gaps remain in the development of rigorous evidence in many key areas. What is known about effective engagement methods, or how to do engagement well? For example, are there engagement approaches and structures most useful based on study approaches and design? How does one define and measure the success of engagement and partnerships, including the ultimate outcomes of engagement, or the impact of engagement? What is the evidence on specific populations and settings, including what established or exploratory methods of engagement are most effective to ensure the inclusion of historically under-represented populations? How could methods for collaboration from other disciplines be uniquely adapted and utilized for engagement and increase the diversity of engagement research partners?

## CONCLUSION

The science of engagement is an emerging paradigm designed to revolutionize the way research is conducted, to empower the communities the research affects most by enabling their partnership in all aspects of the research process. We have learned that engagement affects both the conduct of research and the people involved for the better. Building trust by being trustworthy is critical. Engagement can strengthen community trust in the research process and increases patients’ willingness to participate. It is not an overstatement to say that engagement seeks to fundamentally change how research is designed, conducted, and disseminated. The ultimate goal is to develop research to support informed decision-making for the patients and communities we serve.

As this special supplement demonstrates, current research on engagement is stemming from multiple funders—with PCORI and VA at the vanguard—and includes multiple subject areas, methodological approaches, and outcomes. It is an emerging field with immense national activity.

Further efforts are needed to help strengthen a community of engagement researchers and methodologists, and this must include the individuals and members of communities most affected by the issues to be studied. Additional funding is needed to help promote novel designs, better measures, and tools, as well as information on the best outcomes to measure. Finally, just as there is increased recognition that patients and stakeholders should not be the object of clinical and other health research, collaboration, and partnership in all aspects of the research process must also be extended to the study of engagement in research and developing the evidence in this field.

## References

[1] Further Consolidated Appropriations Act, 2020, Public Law No: 116-94, 116^th^ Cong., Sec. 104, (2019).

[2] Sheridan S, Schrandt S, Forsythe L, Hilliard TS, Paez KA (2017). Advisory Panel on Patient Engagement (2013 inaugural panel). *The PCORI engagement rubric: promising practices for partnering in research*. Ann Fam Med..

[3] Knight SJ, Haibach JP, Hamilton AB, et al. Veteran engagement in health services research: a conceptual model. [SPI 7309] 10.1007/s11606-021-07309-z PMC8993964 35349018

[4] Nugent SM, Cottrell E, Knight SJ, Helfand M. Health experience research as a mechanism for veteran engagement in VA healthcare research and care delivery. [SPI 7306] 10.1007/s11606-021-07306-2 PMC8993951 35349029

[5] Fletcher EH, Gabrielian S, Brown L, et al. Lessons Learned by Collaborating with Structurally Vulnerable Veterans via a Veterans Engagement Group. [SPI 7075] 10.1007/s11606-021-07075-y PMC8960678 35349015

[6] Bowen D, Ackermann N, Thompson V, et al. A study examining the usefulness of a new measure of research engagement. [SPI 6993] 10.1007/s11606-021-06993-1 PMC8960689 35349011

[7] Martinez J, Piersol CV, Lucas KA, Leland NE. Operationalizing Stakeholder Engagement Through the Stakeholder-Centric Engagement Charter (SCEC). [SPI 7029] 10.1007/s11606-021-07029-4 PMC8994015 35349021

[8] Mardian A, Perez L, Pun T, et al. Engagement in Prescription Opioid Tapering Research: The EMPOWER Study and a Coproduction Model of Success. [SPI 7085] 10.1007/s11606-021-07085-w PMC8993995 34389937

[9] Merker VL, Hyde JK, Herbst A, et al. Evaluating the Impacts of Patient Engagement on Health Services Research Teams: Lessons from the Veteran Consulting Network. [SPI 6987] 10.1007/s11606-021-06987-z PMC8993982 35349028

[10] Nearing KA, Adams HM, Alsphaugh J, et al. Engaging the wisdom of older Veterans to enhance VA healthcare, research and services. [SPI 7076] 10.1007/s11606-021-07076-x PMC8960672 35349016

[11] Ho EY, Agne R, Santana T, et al. A Communication Perspective on What Patient Advisory Boards Do: Action-Implicative Discourse Analysis and Negotiating Advice. [SPI 7062] 10.1007/s11606-021-07062-3 PMC8993984 34591265

[12] Chrystal JG, Dyer KE, Gammage CE, et al. Increasing Engagement of Women Veterans in Health Research. [SPI 7126] 10.1007/s11606-021-07126-4 PMC8993961 35349014

[13] Maurer M, Mangrum R, Hilliard-Booner T, et al. Understanding the Influence and Impact of Stakeholder Engagement in Patient-centered Outcomes Research: A Qualitative Study. [SPI 7104] 10.1007/s11606-021-07104-w PMC8993962 35349017

[14] McNeal DM, Fehling K, Ho PM, et al. Engaging Stakeholders in Identifying Access Research Priorities for the Department of Veterans Affairs. [SPI 7195] 10.1007/s11606-021-07195-5 PMC8993958 35349024

[15] van Eeghen C, Hitt JR, Pomeroy DJ, et al. Co-creating the Patient Partner Guide by a Multiple Chronic Conditions Team of Patients, Clinicians, and Researchers: Observational Report. [SPI 7308] 10.1007/s11606-021-07308-0 PMC8960693 35349025

